# Supplementary material for: A Likelihood Approach for Real-Time Calibration of Stochastic Compartmental Epidemic Models
Source: PLoS Comput Biol. 2017 Jan 17;13(1):e1005257. doi: 10.1371/journal.pcbi.1005257 (PMC5240920; doi:10.1371/journal.pcbi.1005257)
Supplement: S1 File — (TAR.GZ) [file pcbi.1005257.s014.tar.gz › HSPH_Online-SI-Revision/IPoi09/n10-mild/n10-mild_BENCH_table-8.pdf]

1	R0	{1.25046, 6.80278}	{1.75035, 12.5893}	{{1.39507, 7.40677}, {2.00049, 16.748}}	{39.9766, 85.7184}	inf	59.	170.636	{27., 394.}	212.173	0.479453	newly	76.	112.364	{20., 267.}	90.3708	0.348265	Reff	1.22245	1.7267	{1.33482, 2.01464}	41.2495	ar	3516.	7039.84	{5197., 8072.}	104.559
2	R0	{1.32013, 4.45847}	{2.42593, 13.2096}	{{1.88486, 8.39294}, {2.96398, 17.7348}}	{83.7646, 196.378}	inf	43.	144.864	{15., 332.}	260.782	0.495023	newly	76.	121.773	{11., 300.}	111.583	0.407444	Reff	1.30046	2.35542	{1.74519, 2.8784}	81.138	ar	4420.	8582.83	{7505., 9363.}	94.1817
3	R0	{1.31785, 5.36796}	{1.54954, 2.94968}	{{1.27121, 1.91565}, {1.65456, 3.37285}}	{18.2888, 45.3533}	inf	49.	277.844	{4., 612.}	494.875	0.907762	newly	66.	866.111	{12., 1616.}	1235.79	1.237	Reff	1.29953	1.47238	{1.2027, 1.86919}	16.8188	ar	4942.	5661.6	{3433., 8078.}	34.7722
4	R0	{1.38801, 6.16031}	{1.54936, 6.74265}	{{1.29927, 4.21286}, {2.11132, 12.644}}	{16.091, 41.6041}	inf	59.	195.356	{13., 486.}	255.443	0.593402	newly	85.	234.4	{21., 542.}	200.444	0.562319	Reff	1.35636	1.54465	{1.20892, 2.17772}	20.5513	ar	4790.	5861.47	{3934., 8564.}	37.2508
5	R0	{1.27955, 11.57}	{1.40027, 12.6364}	{{1.39322, 12.5555}, {1.40677, 12.6439}}	{9.43452, 9.65432}	inf	102.	362.028	{77., 530.}	265.714	0.559539	newly	64.	200.556	{48., 291.}	223.611	0.519832	Reff	1.19011	1.08616	{0.899045, 1.3467}	14.3082	ar	3460.	4957.5	{4739., 5365.}	48.7139
6	R0	{1.22184, 4.34395}	{1.96232, 4.68769}	{{1.31246, 2.23442}, {2.77394, 7.74418}}	{60.6518, 39.1809}	inf	20.	109.593	{3., 293.}	476.296	0.783706	newly	28.	311.333	{12., 731.}	1034.66	1.03116	Reff	1.21683	2.07548	{1.33421, 2.78318}	70.5642	ar	3524.	7050.2	{4013., 9301.}	118.518
7	R0	{1.23856, 4.59068}	{1.46318, 4.21684}	{{1.31023, 3.04996}, {1.69756, 6.15907}}	{18.1359, 25.38}	inf	52.	228.205	{11., 474.}	363.807	0.677594	newly	78.	437.231	{23., 912.}	480.013	0.770305	Reff	1.20995	1.41113	{1.15954, 1.92249}	18.1693	ar	3855.	5535.56	{4214., 8042.}	53.7138
8	R0	{1.34531, 10.6689}	{1.25906, 3.86923}	{{1.25656, 3.82585}, {1.25656, 3.82585}}	{6.41088, 63.7335}	inf	98.	172.75	{67., 255.}	85.3571	0.249855	newly	72.	313.7	{147., 435.}	335.694	0.604997	Reff	1.29352	1.04904	{0.911132, 1.16408}	18.9003	ar	4852.	3723.6	{3033., 4061.}	23.2564
9	R0	{1.31775, 5.19226}	{2.31202, 13.9412}	{{1.85533, 9.28761}, {2.8568, 18.33}}	{75.4523, 169.066}	inf	51.	156.848	{19., 366.}	230.6	0.504881	newly	85.	111.121	{14., 216.}	85.3832	0.407702	Reff	1.29522	2.28188	{1.7918, 2.72641}	76.1773	ar	4708.	8400.06	{7471., 9283.}	81.4487
10	R0	{1.25916, 6.89207}	{1.31535, 3.47086}	{{1.31535, 3.47086}, {1.31535, 3.47086}}	{4.4625, 49.6398}	inf	121.	244.375	{82., 337.}	117.149	0.369642	newly	119.	461.938	{190., 624.}	296.481	0.586653	Reff	1.17404	0.999255	{0.825777, 1.25484}	17.2318	ar	3823.	4484.75	{4051., 4851.}	17.3097
11	R0	{1.20377, 5.46566}	{1.37988, 2.86109}	{{1.37363, 2.81014}, {1.43579, 3.38828}}	{14.63, 47.6534}	inf	61.	206.5	{69., 410.}	250.252	0.517818	newly	77.	616.385	{200., 1115.}	705.395	0.837193	Reff	1.17392	1.24931	{1.11525, 1.37707}	10.4789	ar	3114.	4905.31	{4213., 5548.}	57.5243
12	R0	{1.27878, 2.55804}	{2.30155, 7.77259}	{{1.53501, 3.72349}, {2.85038, 10.6536}}	{80.1147, 204.274}	inf	6.	53.7424	{9., 116.}	803.283	0.828193	newly	12.	88.9091	{15., 188.}	647.98	0.738636	Reff	1.27328	2.36473	{1.62809, 2.84236}	85.7194	ar	3930.	8358.38	{6574., 9321.}	115.706
13	R0	{1.41227, 5.45696}	{1.31228, 5.83021}	{{1.20105, 3.78882}, {1.39507, 7.40677}}	{7.87358, 20.1839}	inf	41.	135.125	{25., 342.}	245.224	0.446725	newly	62.	160.792	{37., 411.}	176.815	0.393758	Reff	1.38233	1.24879	{1.14662, 1.41086}	11.9653	ar	4822.	4615.21	{3638., 5515.}	13.9595
14	R0	{1.32917, 7.27593}	{1.49323, 7.23502}	{{1.34002, 5.27199}, {1.76659, 10.896}}	{12.5617, 31.0585}	inf	80.	345.25	{51., 629.}	346.797	0.598023	newly	96.	343.813	{94., 571.}	267.839	0.529115	Reff	1.28969	1.3512	{1.13008, 1.66766}	14.3962	ar	4758.	5806.69	{4579., 7351.}	26.0259
15	R0	{1.31629, 9.97481}	{1.54241, 6.75648}	{{1.28596, 3.98513}, {2.11132, 12.644}}	{18.6377, 44.3625}	inf	84.	327.757	{39., 663.}	309.492	0.637717	newly	70.	382.514	{53., 677.}	460.656	0.76204	Reff	1.2843	1.37653	{1.05952, 1.79029}	19.4805	ar	4543.	5656.62	{3934., 8255.}	34.0435
16	R0	{1.36735, 5.74166}	{1.25198, 3.23358}	{{1.22544, 3.07157}, {1.26656, 3.29313}}	{8.43753, 43.6821}	inf	40.	88.	{4., 190.}	169.038	0.661656	newly	65.	217.154	{6., 469.}	277.988	0.791306	Reff	1.34356	1.19342	{1.11221, 1.25174}	11.1837	ar	4516.	3084.	{98., 4228.}	32.7008
17	R0	{1.27524, 13.2396}	{1.64502, 14.9009}	{{1.34091, 8.76637}, {1.86254, 19.0665}}	{29.2135, 26.3235}	inf	39.	43.2128	{7., 114.}	72.2313	0.346387	newly	23.	24.4043	{3., 54.}	73.4505	0.403736	Reff	1.26504	1.6518	{1.38199, 1.87069}	31.2398	ar	3731.	6524.94	{4647., 7620.}	79.0633
18	R0	{1.21884, 7.71545}	{1.23312, 3.22444}	{{1.22544, 3.07157}, {1.25656, 3.82585}}	{1.17134, 58.208}	inf	68.	138.889	{50., 239.}	117.32	0.305031	newly	61.	277.556	{108., 425.}	355.009	0.599391	Reff	1.17923	1.00473	{0.823741, 1.21296}	15.5075	ar	3080.	3556.39	{2960., 4142.}	16.9102
19	R0	{1.34879, 6.71464}	{1.33649, 3.24859}	{{1.31023, 3.04996}, {1.42949, 4.01966}}	{3.49087, 51.6193}	inf	96.	247.12	{103., 436.}	163.75	0.38496	newly	111.	554.12	{242., 831.}	399.568	0.656325	Reff	1.29578	1.17787	{1.03796, 1.32128}	10.4128	ar	4585.	4694.12	{4148., 5534.}	11.0848
20	R0	{1.37284, 5.75036}	{1.12242, 1.78722}	{{1.12171, 1.78492}, {1.12171, 1.78492}}	{18.2411, 68.9199}	inf	46.	32.7273	{4., 97.}	71.5415	0.633038	newly	83.	143.909	{32., 386.}	115.444	0.517072	Reff	1.34758	1.06819	{1.0101, 1.10141}	20.7325	ar	4750.	1827.55	{1001., 2664.}	61.5254
21	R0	{1.29938, 5.36419}	{1.96041, 9.43669}	{{1.31535, 3.47086}, {2.74988, 16.2348}}	{51.3314, 87.7367}	inf	36.	120.571	{14., 279.}	262.996	0.57463	newly	59.	128.054	{7., 288.}	163.347	0.541887	Reff	1.28145	2.0907	{1.39383, 2.84811}	63.4196	ar	4337.	7577.91	{5497., 9274.}	82.2717
22	R0	{1.31882, 7.47626}	{1.61352, 9.14791}	{{1.24719, 4.06287}, {2.01498, 15.0173}}	{23.6534, 49.5489}	inf	67.	197.651	{30., 426.}	218.674	0.501722	newly	68.	202.233	{21., 468.}	223.666	0.486222	Reff	1.28479	1.57088	{1.10284, 2.07741}	29.1984	ar	3977.	6410.49	{4362., 8391.}	61.7738
23	R0	{1.26215, 3.46375}	{1.22517, 3.53297}	{{1.06836, 1.81005}, {1.61188, 8.7843}}	{17.6228, 59.401}	inf	5.	45.5652	{8., 107.}	811.304	0.780089	newly	32.	96.4783	{11., 197.}	234.375	0.448582	Reff	1.25672	1.40905	{1.01134, 2.07919}	28.2152	ar	3607.	3971.35	{487., 8309.}	72.475
24	R0	{1.16712, 4.19464}	{1.98564, 12.8089}	{{1.67732, 9.44073}, {2.24242, 15.7579}}	{70.1319, 205.364}	inf	63.	335.308	{52., 645.}	446.581	0.675143	newly	109.	240.827	{35., 446.}	156.122	0.469177	Reff	1.13853	1.87201	{1.58845, 2.14555}	64.9822	ar	3000.	7741.75	{7275., 8657.}	161.884
25	R0	{1.22751, 3.34517}	{2.22471, 10.2649}	{{1.42659, 4.00571}, {2.87181, 15.1849}}	{82.3964, 209.909}	inf	2.	8.12698	{1., 18.}	322.222	0.539236	newly	9.	10.9841	{0., 27.}	111.64	0.615063	Reff	1.22628	2.32899	{1.53986, 2.87037}	89.9226	ar	3086.	7399.97	{37., 9265.}	165.087
26	R0	{1.18747, 5.88083}	{1.35965, 4.48414}	{{1.35758, 4.45823}, {1.35758, 4.45823}}	{14.4998, 23.7499}	inf	134.	314.808	{157., 450.}	141.418	0.374722	newly	150.	465.923	{242., 633.}	215.128	0.484936	Reff	1.11017	0.993848	{0.819164, 1.18517}	15.2305	ar	3614.	4830.08	{4534., 5103.}	33.6491
27	R0	{1.29023, 4.76202}	{1.24201, 3.70905}	{{1.22516, 3.07157}, {1.25656, 3.82585}}	{3.73697, 22.1118}	inf	32.	120.682	{37., 217.}	278.551	0.514401	newly	65.	251.182	{106., 422.}	286.434	0.53632	Reff	1.26391	1.12859	{1.01216, 1.20831}	10.7066	ar	4245.	3478.09	{3156., 3792.}	18.0662
28	R0	{1.2258, 12.9056}	{1.45462, 13.5677}	{{1.45653, 13.6021}, {1.45653, 13.6021}}	{18.6668, 5.13025}	inf	157.	369.875	{212., 515.}	141.361	0.371428	newly	86.	186.75	{112., 262.}	124.128	0.340716	Reff	1.08814	0.912131	{0.726517, 1.17484}	21.1906	ar	3639.	5492.13	{5219., 5776.}	50.924
29	R0	{1.34702, 6.29049}	{2.04767, 12.6018}	{{1.71337, 8.80249}, {2.315, 15.4658}}	{52.1566, 101.923}	inf	78.	175.519	{27., 401.}	155.271	0.391794	newly	109.	132.352	{31., 299.}	82.7897	0.373269	Reff	1.31765	1.99832	{1.61331, 2.26524}	51.9747	ar	4560.	7983.59	{6929., 8658.}	75.3062
30	R0	{1.37931, 6.78741}	{1.2589, 2.98808}	{{1.24751, 2.5364}, {1.26656, 3.29313}}	{8.72981, 55.9761}	inf	121.	168.5	{40., 275.}	68.0316	0.278575	newly	153.	382.409	{91., 591.}	167.528	0.430491	Reff	1.31324	1.00465	{0.808636, 1.14421}	23.4982	ar	4667.	3748.32	{3315., 4232.}	19.6846
31	R0	{1.16062, 4.47529}	{2.2908, 6.06131}	{{1.61068, 3.12918}, {2.82914, 8.62388}}	{97.3769, 46.1875}	inf	8.	32.1528	{2., 70.}	327.951	0.637533	newly	12.	83.5278	{5., 186.}	620.139	0.823813	Reff	1.1576	2.3338	{1.79389, 2.82801}	101.606	ar	3037.	7775.81	{4989., 9293.}	172.664
32	R0	{1.37647, 18.7122}	{1.59419, 15.5715}	{{1.59192, 15.4649}, {1.59192, 15.4649}}	{15.8171, 16.7843}	inf	284.	603.265	{397., 813.}	115.752	0.318137	newly	111.	264.324	{164., 359.}	139.083	0.355689	Reff	1.17055	0.96765	{0.69933, 1.37319}	24.6635	ar	4824.	6417.82	{6180., 6610.}	33.0395
33	R0	{1.35084, 6.08841}	{1.23474, 3.884}	{{1.21159, 3.57392}, {1.25656, 4.06287}}	{8.59464, 36.2067}	inf	31.	91.8333	{14., 183.}	215.323	0.482823	newly	44.	189.083	{16., 387.}	346.97	0.616453	Reff	1.3326	1.17069	{1.10289, 1.22967}	12.15	ar	4759.	3213.96	{2888., 3792.}	32.4657
34	R0	{1.36912, 6.316}	{1.29988, 3.78419}	{{1.25474, 3.26445}, {1.35758, 4.45823}}	{5.54496, 40.0857}	inf	47.	181.04	{14., 365.}	309.021	0.662456	newly	61.	352.72	{44., 603.}	493.836	0.782868	Reff	1.33681	1.16351	{0.988986, 1.29282}	13.0161	ar	4537.	4003.68	{2934., 5038.}	18.9385
35	R0	{1.35831, 7.09038}	{1.55659, 6.44006}	{{1.25474, 3.26445}, {1.98131, 11.1772}}	{16.9745, 40.133}	inf	102.	344.85	{105., 633.}	247.892	0.521419	newly	115.	429.	{129., 735.}	281.87	0.568557	Reff	1.31498	1.40788	{1.03064, 1.89952}	19.8142	ar	4667.	5953.	{3992., 8205.}	35.8174
36	R0	{1.28315, 5.89938}	{2.26394, 11.491}	{{1.739, 7.10524}, {2.76203, 15.6124}}	{76.4361, 95.9008}	inf	88.	196.108	{33., 484.}	152.185	0.382647	newly	139.	169.615	{18., 391.}	85.5119	0.395095	Reff	1.25672	2.28894	{1.75741, 2.72807}	82.1365	ar	4460.	8559.22	{7505., 9273.}	91.9107
37	R0	{1.19303, 2.75113}	{1.28485, 1.9981}	{{1.27121, 1.91565}, {1.31246, 2.23442}}	{7.69607, 27.6466}	inf	7.	63.3448	{0., 136.}	837.438	1.02388	newly	22.	324.586	{0., 626.}	1408.93	1.29093	Reff	1.1848	1.24838	{1.18795, 1.295}	5.73769	ar	3142.	3387.24	{49., 4571.}	41.9961
38	R0	{1.36033, 7.8088}	{1.41356, 4.64846}	{{1.31535, 3.47086}, {1.47651, 5.4396}}	{6.14608, 42.1927}	inf	85.	281.75	{21., 542.}	250.21	0.607049	newly	88.	461.464	{33., 811.}	441.518	0.78186	Reff	1.32551	1.2693	{1.02139, 1.45968}	13.608	ar	4941.	5081.5	{4237., 5862.}	15.8759
39	R0	{1.27353, 3.70845}	{1.19051, 2.00776}	{{1.19264, 2.01465}, {1.19264, 2.01465}}	{6.51872, 45.8599}	inf	37.	141.077	{55., 194.}	290.021	0.564656	newly	69.	492.692	{195., 611.}	614.047	0.800239	Reff	1.24908	1.04489	{0.958525, 1.11643}	16.3471	ar	3725.	3346.15	{2980., 3724.}	10.4884
40	R0	{1.19983, 12.1486}	{1.4578, 15.41}	{{1.45513, 15.3398}, {1.45513, 15.3398}}	{21.5005, 26.8461}	inf	164.	428.871	{241., 586.}	167.014	0.40945	newly	86.	198.065	{119., 275.}	136.759	0.365213	Reff	1.07625	1.07844	{0.829424, 1.30714}	14.657	ar	3619.	5516.65	{5233., 5740.}	52.4356
41	R0	{1.20935, 2.67169}	{2.15394, 4.52399}	{{1.4023, 2.07266}, {2.70127, 6.23525}}	{78.1072, 76.138}	inf	13.	65.9104	{3., 167.}	434.099	0.722903	newly	45.	250.015	{5., 642.}	480.929	0.79545	Reff	1.20379	2.2101	{1.47876, 2.82057}	83.596	ar	3429.	7419.42	{5006., 9272.}	134.228
42	R0	{1.23313, 4.74781}	{1.19264, 2.01465}	{{1.19264, 2.01465}, {1.19264, 2.01465}}	{3.28351, 57.5668}	inf	49.	52.1429	{6., 105.}	79.8834	0.418397	newly	79.	157.357	{14., 353.}	154.521	0.44038	Reff	1.18196	0.849765	{0.769372, 1.02961}	28.1052	ar	3835.	3300.	{2700., 3724.}	13.9505
43	R0	{1.26105, 2.39713}	{1.12171, 1.78492}	{{1.12171, 1.78492}, {1.12171, 1.78492}}	{11.0495, 25.5393}	inf	6.	25.8462	{5., 54.}	348.718	0.677907	newly	32.	109.538	{13., 222.}	271.154	0.651359	Reff	1.25298	1.09477	{1.05788, 1.10769}	12.6265	ar	3723.	1376.38	{181., 2565.}	63.0302
44	R0	{1.36948, 8.88006}	{1.27076, 3.69586}	{{1.22544, 3.07157}, {1.35758, 4.45823}}	{7.56575, 58.3802}	inf	75.	117.241	{13., 259.}	104.506	0.462489	newly	68.	244.414	{21., 499.}	286.41	0.624074	Reff	1.33689	1.18336	{1.06907, 1.30526}	11.5799	ar	4437.	3753.72	{2956., 4877.}	19.2838
45	R0	{1.34882, 3.3791}	{1.91498, 11.8662}	{{1.23086, 5.61163}, {2.70836, 18.727}}	{46.6011, 251.231}	inf	2.	1.82	{0., 4.}	83.	0.572355	newly	4.	1.81	{0., 4.}	83.25	0.971541	Reff	1.34828	1.91441	{1.23074, 2.70809}	46.6106	ar	4422.	3415.5	{1., 9028.}	91.6748
46	R0	{1.23774, 4.34917}	{2.17494, 7.39909}	{{1.43579, 3.38828}, {2.74056, 10.6768}}	{75.7183, 77.8194}	inf	31.	157.19	{42., 314.}	413.737	0.62248	newly	58.	258.552	{46., 586.}	354.637	0.565552	Reff	1.22252	2.22932	{1.54924, 2.74606}	82.3968	ar	3685.	8304.64	{6578., 9296.}	125.363
47	R0	{1.34327, 4.78811}	{2.10265, 8.90803}	{{1.31535, 3.47086}, {2.73634, 13.2503}}	{57.5307, 95.9658}	inf	9.	56.0185	{3., 138.}	543.416	0.777605	newly	13.	63.7222	{4., 139.}	412.678	0.70249	Reff	1.33642	2.26046	{1.54355, 2.81195}	69.316	ar	4517.	7739.15	{5631., 9273.}	86.1347
48	R0	{1.24366, 4.25814}	{1.10488, 2.27109}	{{1.09005, 2.14605}, {1.12171, 2.14605}}	{11.1589, 46.6646}	inf	15.	62.6667	{5., 139.}	341.111	0.582094	newly	19.	185.5	{9., 313.}	896.491	0.874323	Reff	1.23073	1.04195	{0.926979, 1.15851}	15.3383	ar	3678.	1861.92	{227., 3185.}	49.3769
49	R0	{1.36017, 7.5415}	{1.35598, 4.45013}	{{1.35758, 4.45823}, {1.35758, 4.45823}}	{0.308344, 40.9915}	inf	84.	319.615	{167., 450.}	280.495	0.556152	newly	104.	474.154	{276., 633.}	355.917	0.639421	Reff	1.284	0.971089	{0.797339, 1.18109}	24.4351	ar	4178.	4813.27	{4534., 5083.}	15.2051
50	R0	{1.25333, 6.63578}	{1.3142, 3.44212}	{{1.31535, 3.47086}, {1.31535, 3.47086}}	{4.85644, 48.1279}	inf	101.	194.125	{45., 317.}	106.436	0.312157	newly	117.	347.625	{110., 601.}	202.244	0.43659	Reff	1.14855	0.889826	{0.733176, 1.19763}	24.4977	ar	3401.	4484.75	{4051., 4851.}	31.8656
